# Supplementary figures and images for: Lepidopteran HMG-CoA reductase is a potential selective target for pest control
Source: PeerJ. 2017 Jan 19;5:e2881. doi: 10.7717/peerj.2881 (PMC5251934; doi:10.7717/peerj.2881)

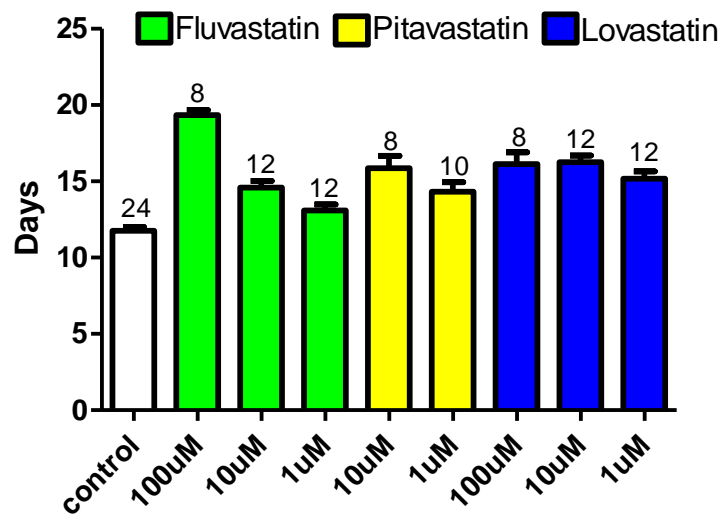

**Figure S2.** The number of days from newly hatched larvae to the 5<sup>th</sup> stadium.

Supplement: Figure S2 [file peerj-05-2881-s002.pdf]
