# Supplementary material for: Lepidopteran HMG-CoA reductase is a potential selective target for pest control
Source: PeerJ. 2017 Jan 19;5:e2881. doi: 10.7717/peerj.2881 (PMC5251934; doi:10.7717/peerj.2881)
Supplement: Figure S1 — The most favoured regions are coloured in red. Allowed, generously allowed, and disallowed regions are indicated as yellow, light yellow, and white regions. [file peerj-05-2881-s001.pdf]

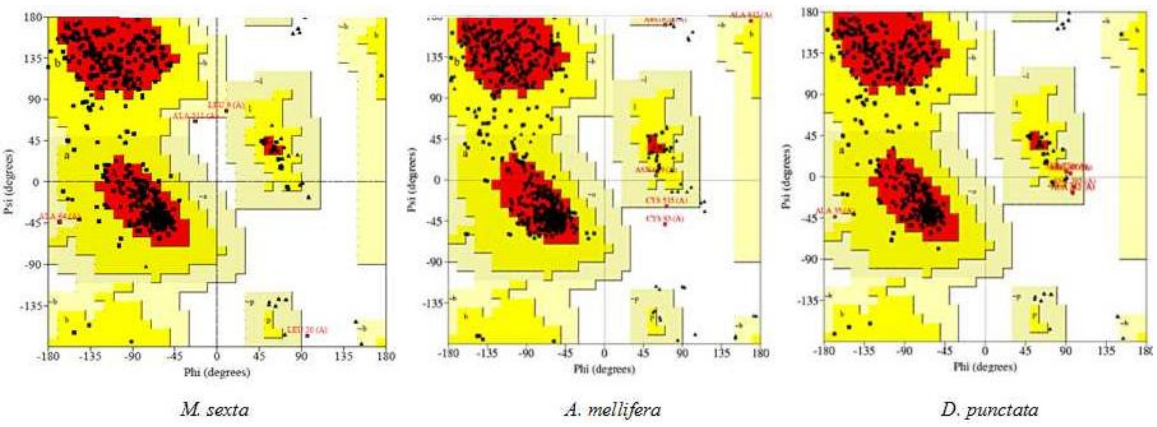

Figure S1. Ramachandran plot of the HMGR models of *M. sexta*, *A. mellifera* and *D. punctata*. The most favoured regions are coloured in red. Allowed, generously allowed, and disallowed regions are indicated as yellow, light yellow, and white regions, respectively.
